# An Experimental and Theoretical Study on the Electrical-Resistance-Based Gage Factor of a Single Carbon Fiber in the Thermal–Mechanical Coupling Effect

**DOI:** 10.3390/ma19091697

**Published:** 2026-04-23

**Authors:** Shiquan Li, Yu Chen, Haojie Wang

**Affiliations:** 1 Taizhou Institute of Science and Technology, Nanjing University of Science and Technology, Taizhou 225300, China; 2 Faculty of Civil Engineering and Mechanics, Jiangsu University, Zhenjiang 212013, China; chenyu_ujs@163.com; 3 Tianping College of Suzhou University of Science and Technology, Suzhou 215009, China; wanghaojie729@163.com

**Keywords:** single carbon fiber, electrical-resistance-based, thermal–mechanical coupling, gage factor (GF), strain, quasi-static, piezoresistive effect

## Abstract

Self-sensing refers to structural material sensing by auxiliary devices without intelligent features. The analysis of the electrical parameters of a single carbon fiber is the foundation of CFRP self-sensing. Focusing on electrical-resistance-based strain, this study conducts a theoretical analysis of the electrical parameters of a single carbon fiber. The relationship between stress-induced strain and resistance is established, yielding the gage factor (GF) under the load effect. Drawing upon the impurity scattering mechanism, the relationship between thermal-induced strain and resistance is formulated, leading to the GF under thermal effects. According to the quasi-static equivalent superposition principle, strain vs. resistance in the effect of thermal–mechanical coupling was established, and a GF model is proposed. The analysis of a single carbon fiber demonstrates that under load effect the contribution of the piezoresistive effect reaches 13.4%, which is non-negligible. Thermal-resistance tests were conducted on a single carbon fiber with different initial states. The thermal-resistance analysis indicated that the resistance of a single carbon fiber decreased with an increase in temperature. The initial state had a significant impact on the GF. The thermal resistance of a free single carbon fiber can be expressed by two types of models, each with an error of less than 0.2% from 223 K to 473 K. Based on four-point bending specimens, the force-resistance test of a single carbon fiber was conducted indirectly. The improvement in the production process has led to an increase in the graphitization degree of carbon fibers. The $K_{S}^{F}$ values of A3 and B3 are 1.411 and 1.405, respectively, both of which are higher than those of carbon fibers in the earlier literature. The strain-resistance analysis showed that the stress-induced GF of a single carbon fiber is lower than the thermal-induced GF. When the deformation was constrained, the stress-induced GF of the single carbon fiber was reduced. Together, the thermal and mechanical properties of a single carbon fiber make it more suitable as a temperature sensor than as a damage sensor.

## 1. Introduction

Electrical-resistance-based strain/damage self-sensing in cement-based materials emerged in 1990s, and the field has grown greatly during the last decade [1,2]. Carbon-Fiber-Reinforced Polymers (CFRPs) exhibit numerous advantages, including high specific strength, high specific modulus, superior thermal conductivity, excellent thermal stability, fatigue resistance, reliable electrical conductivity, a low coefficient of thermal expansion, non-magnetic properties, and favorable machinability. As such, it is regarded as a strategic material in high-performance structural and engineering applications. Theoretical research on the manufacturing, processing, self-sensing, and self-monitoring of CFRPs has emerged as a focal point of investigation across various industries [3,4]. Over the past decade, research on the self-sensing capabilities of CFRPs has gained incremental recognition [5,6,7]. The main reason for this incremental recognition gain is that carbon fibers and carbon fiber composites can achieve self-sensing through resistance measurement without adding any additional conductive materials. Research on self-sensing has facilitated the integrated application of CFRPs in high-load-bearing and self-monitoring functionalities. For example, in 2025 the Changtai Yangtze River Bridge, featuring a main span of 1208 m, was constructed in Jiangsu Province of China. As part of this project, the CFRP cable-based Thermal-Adapting Tower-Deck Restraint System (TARS) was implemented to eliminate the influence of thermal deformation of the main girder on the internal forces of the structure, thereby achieving complete thermal self-adaptation of the tower-deck constraint system [8].

However, existing research is yet to fully elucidate the conductive mechanism of carbon fibers, and the models for resistance and the GFs of single carbon filaments under coupled effects require further in-depth investigation [9,10]. The pertinent research areas include the following: (1) The established conductive theory of carbon fibers fails to adequately account for their piezoresistive effects. (2) The temperature ranges investigated in studies on the temperature-dependent resistivity of carbon fibers are excessively limited. (3) The theoretical framework describing the electrical resistance of single carbon fibers under thermal–mechanical coupling remains inadequately elucidated.

As a fundamental component of CFRPs, it is essential to analyze the electrical conduction theory and parameters of individual carbon fibers and to clarify the effects of thermal–mechanical coupling. The relevant literature is poised to advance the research on self-sensing capabilities of CFRPs and facilitate the self-monitoring of structural systems. Previous research findings [11] indicate that mechanical and thermal effects induce geometric deformation (including linear strain, areal strain, and volumetric strain) in carbon fibers, whereas moisture exposure does not. This study conducts an in-depth analysis of the conductive mechanisms of carbon fiber, derives the theoretical model for the resistance of a single carbon fiber (including stress-induced, thermal-induced, and thermal–mechanical coupling effects), and establishes the corresponding theoretical model of the GF. In this study, tests were conducted using PAN-based continuous carbon fibers. Considering that the resistance of a single carbon fiber is at the kilo-ohm level, which is much greater than the contact resistance of the electrode, measurement of the resistivity of the fiber was conducted using the two-probe method [12]. Thermal-resistance tests and force-resistance tests were conducted on different forms of single carbon fibers, and corresponding analyses were carried out.

## 2. Theoretical Analysis

**Figure 1:**
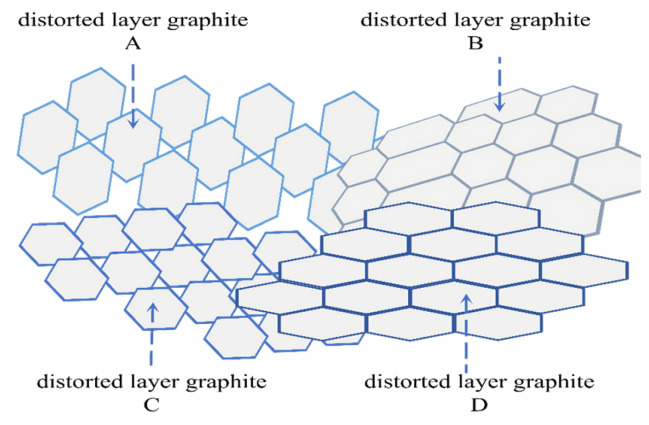
Distorted layer graphite structure.

**Figure 2:**
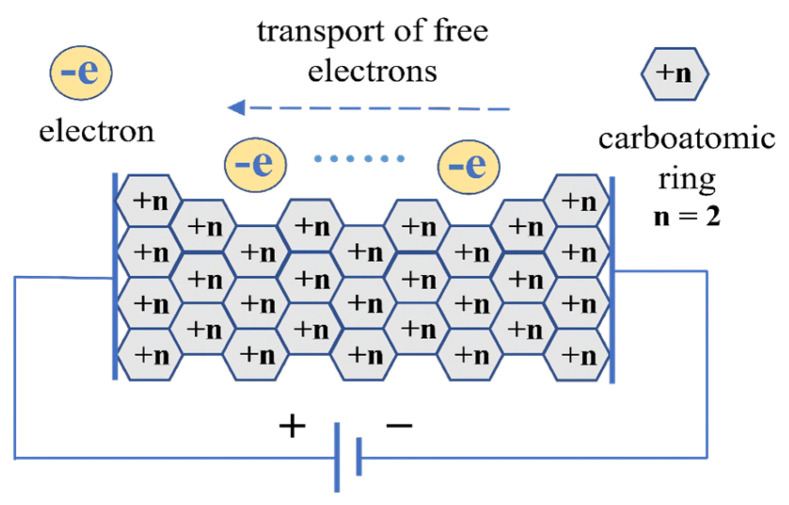
Graphite lamella conductive model.

Figure 1 shows the distorted layer graphite structure in carbon fiber. Figure 2 shows the graphite lamella conductive model in carbon fiber [13]. The carbon content reaches 95% in carbon fibers, with nitrogen as the main impurity, which originates from g-C_3_N_4_ [14,15]. The bandgap width of g-C_3_N_4_ is 2.7 eV, which means it is a negative semiconductor [16]. g-C_3_N_4_ in the impurity components makes the conductivity of carbon fibers approach that of semiconductors. The research results of Han [12] and Huang [15] support this conclusion.

**Figure 3:**
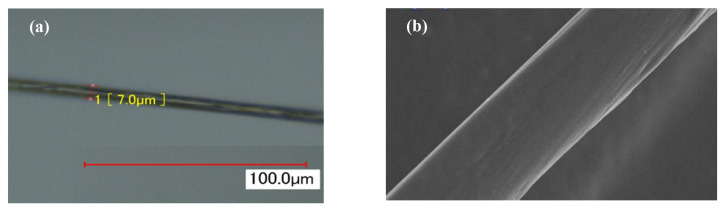
Single carbon fiber: (**a**) ultra depth of field image and (**b**) SEM image.

The analysis of the electrical parameters of a single carbon fiber filament is the foundation of CFRP self-sensing. Figure 3 shows that a single carbon fiber is a cylinder with a smooth surface. Due to the preferred orientation of carbon layers along the fiber axis and the anisotropic nature of carbon fiber, higher electrical conductivity and elastic modulus values exist in the fiber axis rather than in the transverse direction [17,18]. Previous research [19,20] shows the transverse effect at the ends of the sensitive grid reduce the GF of the strain gauge. Using a single carbon fiber to replace the sensitive grid can eliminate the transverse effect.

According to the Chinese National Standard “Metallic Bonded Resistance Strain Gauges (GB/T 13992)” [21], the sensitivity $S_{n}$ of a single carbon fiber can be given by

(1) $$S_{n}=\frac{\Delta R}{\varepsilon }$$

where ΔR is the change of R, R is the volume resistivity, and ε is the axial strain.

The GF of single carbon fiber $K_{S}$ is given by

(2) $$K_{S}=\frac{\Delta R/R_{0}}{\varepsilon }=\frac{S_{n}}{R_{0}}$$

where $R_{0}$ is the initial volume resistance.

### 2.1. Electrical Parameters

According to the graphite lamella conductive model in Figure 2, the volume resistance of a single carbon fiber R relates to the volume resistivity ρ by the well-known equation

(3) $$R=\rho \frac{L}{A}$$

where L is the length in the direction of the resistance R, and A is the cross-sectional area. Equation (3) assumes that the material is isotropic in the two transverse directions.

Based on Equation (3), the two sides of the equation can be differentiated to obtain Equation (4):

(4) $$\left\{\begin{array}{c} \frac{dR}{R}=\frac{d\rho }{\rho }+\frac{dL}{L}-\frac{dA}{A}\\ \frac{dL}{L}=\varepsilon \\ \frac{dA}{A}=2\frac{dr}{r}=2\varepsilon_{r} \end{array}\right.$$

where $\frac{d\rho }{\rho }$ is the piezoresistive effect, $\frac{dL}{L}-\frac{dA}{A}$ is the strain effect, and $\varepsilon_{r}$ is the radial strain.

The radial strain $\varepsilon_{r}$ relates to the axial strain ε by Equation (5):

(5) $$\varepsilon_{r}=-\mu \varepsilon$$

where μ is the Poisson’s ratio.

Considering the conductivity of carbon fibers approaches that of semiconductors [22], its resistance change rate of the volume resistivity $\frac{d\rho }{\rho }$ is given by

(6) $$\frac{d\rho }{\rho }=\pi_{f}\sigma =\pi_{f}E\varepsilon$$

where $\pi_{f}$ is the axial piezoresistivity, σ is the axial stress, and E is Young’s modulus.

### 2.2. The GF Theory Model Under the Load Effect (F ≠ 0, ΔT = 0)

According to Equations (3)–(5),

(7) $${\left(\frac{dR}{R}\right)}^{F}=(1+2\mu +\pi_{f}E)\cdot \varepsilon^{F}$$

where $\varepsilon^{F}$ is the axial strain under load, and F notes the effect of load.

Combining Equations (2) and (7),

(8) $$K_{S}^{F}=\frac{{(dR/R)}^{F}}{\varepsilon^{F}}=1+2\mu +\pi_{f}E$$

where $K_{S}^{F}$ is the GF under load, while 1+2μ and $\pi_{f}E$ are respectively derived from the effects of dimension change and resistivity change.

### 2.3. The GF Theory Model Under the Effect of Temperature (F = 0, ΔT ≠ 0)

**Figure 4:**
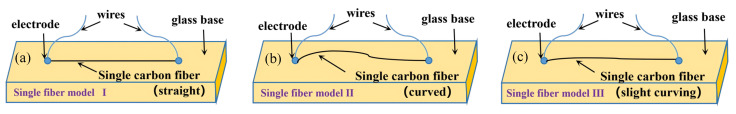
Single carbon fiber models under the effect of temperature: (**a**) straight model; (**b**) curved model; and (**c**) slightly curving model.

Under the effect of temperature, different models of a single carbon fiber are shown in Figure 4. In Model I, the deformation is constrained, and the straight state of the single carbon fiber specimen remains unchanged. In Model II, the deformation is unconstrained, and the curved state of the single carbon fiber remains unchanged. In Model III, with the rise of temperature, the shape of a single carbon fiber will alternate between a straight line and a curve.

For the single carbon fiber with free deformation, shown as the curved model in Figure 4b, the temperature effect on the change rate of resistance includes the strain effect and the piezoresistive effect.

Based on the mechanism of impurity scattering, Zheng et al. [23] obtained the conductivity $\sigma_{f}$ of carbon fiber tow, as shown in Equation (9)

(9) $$\sigma_{f}=nq\upsilon$$

where n is the carrier concentration, q is the single electron charge, and υ is the electron mobility. The electron mobility υ relates to the electron mobility $\upsilon_{0}$ at *T* = 0 K and to temperature by Equation (10)

(10) $$\upsilon =cT^{\frac{3}{2}}+\upsilon_{0}$$

where *c* is a constant parameter, according to reference [24] and semiconductor physics.

Based on Equations (3), (9) and (10), the conductivity of a single carbon fiber is shown by

(11) $$G=\frac{1}{R}=\frac{A}{\rho_{f}L}=\frac{\sigma_{f}A}{L}=\frac{nqA}{L}(cT^{\frac{3}{2}}+\upsilon_{0})=aT^{\frac{3}{2}}+b$$

where $a=\frac{nqcA}{L}$ and $b=\frac{nq\upsilon_{0}A}{L}$.

Then, Equation (11) becomes

(12) $$R=\frac{1}{aT^{\frac{3}{2}}+b}$$

By taking the differential form of Equation (11), Equation (13) is given by

(13) $${\left(\frac{dR}{R}\right)}^{T}=(1+2\mu^{T})\alpha \cdot dT-\frac{dn}{n}-\frac{3}{2}\cdot \frac{T^{1/2}}{T^{3/2}+\upsilon_{0}/c}\cdot dT$$

where $\mu^{T}$ is the equivalent Poisson’s ratio, and T notes the effect of temperature.

$\varepsilon^{T}$ and $\varepsilon_{r}^{T}$ are the axial strain and radial strain under temperature action, respectively.

(14) $$\left\{\begin{array}{c} \varepsilon^{T}=(\frac{dL}{L_{0}}{)}^{T}=\alpha \cdot dT\\ \varepsilon_{r}^{T}=(\frac{dr}{r_{0}}{)}^{T}=\alpha_{r}\cdot dT \end{array}\right.$$

where α<0 and $\alpha_{r}>0$ are the axial and radial thermal expansion coefficients, respectively.

Based on Equations (5) and (14), Equation (15) can be obtained.

(15) $$\mu^{T}=-\frac{\alpha_{r}}{\alpha }$$

When undoped with conductive particles, the carrier concentration in carbon fibers only changes slightly with volume [24]. Then, Equations (16)–(18) can be obtained.

(16) $$-\frac{dn}{n}=\frac{dV}{V}$$

(17) $$\frac{dV}{V}=\bar{\beta }\cdot dT$$

(18) $$\bar{\beta }\approx \alpha +2\alpha_{r}=(1-2\mu^{T})\alpha$$

where $\frac{dn}{n}$, $\frac{dV}{V}$, and $\bar{\beta }$ are the rate of change of carrier concentration, the volumetric change rate, and volumetric strain, respectively.

Based on Equations (15)–(18), Equation (16) becomes

(19) $$-\frac{dn}{n}\approx (1-2\mu^{T})\alpha \cdot dT$$

Substituting Equation (19) into Equation (13), Equation (20) can be formed, combining Equation (14).

(20) $$(\frac{dR}{R}{)}^{T}=2\alpha \cdot dT-\frac{3}{2}\cdot \frac{T^{1/2}}{T^{3/2}+\upsilon_{0}/c}\cdot dT=(2-\frac{3}{2\alpha }\cdot \frac{T^{1/2}}{T^{3/2}+\upsilon_{0}/c})\cdot \varepsilon^{T}$$

According to Equation (8), Equation (21) can be formed.

(21) $$K_{S}^{T}=\frac{(dR/R{)}^{T}}{\varepsilon^{T}}=2-\frac{3}{2\alpha }\cdot \frac{T^{1/2}}{T^{3/2}+\upsilon_{0}/c}$$

where $K_{S}^{T}$ is the GF under the effect of temperature, and $-\frac{3}{2\alpha }\cdot \frac{T^{1/2}}{T^{3/2}+\upsilon_{0}/c}$ is caused by the change in resistivity.

### 2.4. The GF Theory Model Under the Thermal–Mechanical Coupling Effect (F ≠ 0, ΔT ≠ 0)

The quasi-static equivalent superposition theory [25] holds that, in common engineering, the load and temperature effects are without impact, the material volume deformation rate is small, and the influence of deformation work on the temperature field can be ignored. The effects can be calculated independently, and then superimposed in the stress field, achieving the thermal–mechanical coupling effect.

According to the quasi-static equivalent superposition theory, Equation (22) can be obtained by combining Equations (7) and (20).

(22) $$\frac{dR}{R}=(\frac{dR}{R}{)}^{F}+(\frac{dR}{R}{)}^{T}=(1+2\mu +\pi_{f}E)\cdot \varepsilon^{F}+(2-\frac{3}{2\alpha }\cdot \frac{T^{1/2}}{T^{3/2}+\upsilon_{0}/c})\cdot \varepsilon^{T}$$

According to Equation (2) and Equation (22), Equation (23) is given by

(23) $$\left\{\begin{array}{c} K_{S}=\frac{dR/R}{\varepsilon }=(1+2\mu +\pi_{f}E)\cdot \frac{\varepsilon^{F}}{\varepsilon }+(2-\frac{3}{2\alpha }\cdot \frac{T^{1/2}}{T^{3/2}+\upsilon_{0}/c})\frac{\varepsilon^{T}}{\varepsilon }\\ \varepsilon =\varepsilon^{F}+\varepsilon^{T} \end{array}\right.$$

Equation (23) indicates that, under the thermal–mechanical coupling effect, the GF of the single carbon fiber with free deformation, shown as the curved model in Figure 4b, depends on material parameters, geometric dimensions, the load effect, and the temperature effect.

The GF models of a single carbon fiber under different effects are shown in Table 1.

## 3. Materials and Methods

### 3.1. Materials and Instruments

The materials used in this study include SYT49S-12k carbon fiber (Zhongfu Shenying Carbon Fiber Co., Ltd., Lianyungang, Jiangsu, China); T700S-6k carbon fiber (Toray Fibers (Nantong) Co.,Ltd., Nantong, Jiangsu, China); conductive silver paste (Ausbond A528, Shenzhen Ausbond Co., Ltd., Shenzhen, Guangdong, China); clear sheet glass (80 mm × 80 mm × 4 mm; thermal expansion coefficient *α*_g_ = 9.5 × 10^−6^ K^−1^; Nantong Zhenhua Photoelectric Co., Ltd., Nantong, Jiangsu, China); high-temperature wires (the maximum rated temperature is up to 300 °C; CP, (Zhonghang Electronic Measuring Instruments Co., Ltd., Xi’an, Shanxi, China); and strain gauges (BE120-3AA, Zhonghang Electronic Measuring Instruments (Xi’an) Co., Ltd., Xi’an, Shanxi, China). The material parameters of the carbon fiber are shown in Table 2.

The main instruments include a universal testing machine (TH-8201S, Suzhou Top-Hung Machinery Co., Ltd., Suzhou, China); a strain gauge indicator (UT7110Y, Wuhan Youtai Electronic Technology Co., Ltd., Wuhan, China); a constant current resistance tester (CTX2518-16, Changzhou Xinyang Electronic Technology Co., Ltd., Changzhou, China); a digital multimeter (Victory 86E, Shenzhen Victor Hi-tech Co., Ltd., Shenzhen, China); and a constant temperature drying oven (101-OS, Shanghai Techeng Machinery Equipment Co., Ltd., Shanghai, China).

### 3.2. Methods and Procedures

#### 3.2.1. Temperature-Resistance Test Design

**Figure 5:**
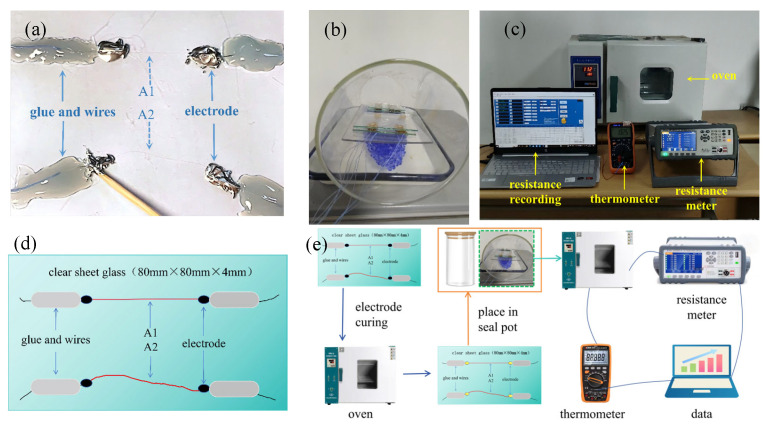
Temperature vs. resistance test of the single carbon fiber specimens: (**a**) a single carbon fiber on glass; (**b**) the closed systems; (**c**) the temperature-resistance test system; (**d**) the specific parameters of the specimen; and (**e**) the specific testing process.

Specimen preparation for the temperature-resistance test involves taking a single carbon fiber from the carbon fiber bundle, placing it on the bottom glass sheet, and adjusting its shape. The conductive silver paste is used to connect the end of the single carbon fiber to the wire, as shown in Figure 5a. The specimen is placed in an oven for heating and curing at 120 °C for 20 min. The electrode clear distance and resistance are tested to confirm the specimen is intact. Four support points are made on the bottom glass and the bottom glass is covered with the top glass to protect the specimen.

The test piece and temperature sensor are placed in the seal pot, as shown in Figure 5b, the wires are led out from the can, and the stopper is installed. The seal pot with the test piece is put into the drying oven. The testing instrument is connected, where the temperature sensor is connected to the multimeter and the computer, as shown in Figure 5c. The drying oven is closed, and the resistance data is recorded after reaching the set temperature. Figure 5d shows the parameters of A1 and A2. Figure 5e shows the specific testing process.

#### 3.2.2. Strain-Resistance Test Design

**Figure 6:**
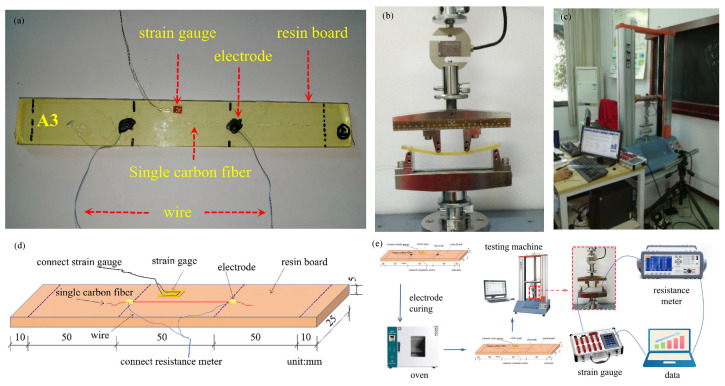
Strain-resistance test of the single carbon fiber: (**a**) the specimen; (**b**) the four-point bending test; (**c**) the test system; (**d**) the specific parameters of A3 and B3; and (**e**) the specific testing process.

During specimen preparation, the loading points and support points are marked on the resin board with a spacing of 50 mm between adjacent points. A single carbon fiber filament is placed on one side of the resin board and adjusted to the desired shape, after which its ends are connected to wires using conductive adhesive, as shown in Figure 6a. The specimen is cured in the oven at 120 °C for 20 min, followed by measurement and recording of the electrode distance and resistance. A strain gauge is attached to the tensile side of the four-point bending plate.

The loading points of the testing machine are adjusted, the specimen is positioned such that the single carbon fiber is placed on the tensile side, and the corresponding wires are connected. A quasi-static load is applied at a rate of 0.5 mm/min (*T*_0_ = 293 K, *F*_0_ = 0 N), as shown in Figure 6b. The loading process is terminated when the single carbon fiber filament fractures and no reading is displayed on the multimeter, with the complete test setup presented in Figure 6c. Figure 6d shows the parameters of A3 and B3. Figure 6e shows the specific testing process.

## 4. Results and Discussion

### 4.1. Temperature-Resistance Test

According to the Chinese National Standard “Determination of volume resistivity of carbon fiber (GB/T 32993)” [26], the volume resistivity of a single carbon fiber can be shown by

(24) $$\rho =\frac{\pi D^{2}R}{4L}$$

where D is the diameter of a single carbon fiber.

According to Equation (24), the resistivity of a single carbon fiber is shown in Table 3. In Table 3, the volume resistivity of a single carbon fiber is calculated based on the parameters of A1 and B1. Based on this, the fiber lengths of A2 and B2 between the electrodes can be calculated, as shown in Table 3. The single carbon fiber volume resistivity of A1 is 1.36 × 10^−5^ Ω·m, less than B1, which is 1.52 × 10^−5^ Ω·m. The volume resistivity of A1 and B1 are both lower than the value provided by the manufacturer in Table 2.

It should be emphasized here that the surface of the carbon fiber has residual oiling components from the production process. The corresponding oil components will gradually volatilize, which will have a certain impact on the electrical resistivity of the fiber. This is the reason why the volume resistivity obtained from the actual test in Table 3 is lower than the value provided by the manufacturer. It is evident that the production process, storage environment, etc. have an impact on the resistivity of carbon fiber.

### 4.2. Temperature-Resistance Analysis

**Figure 7:**
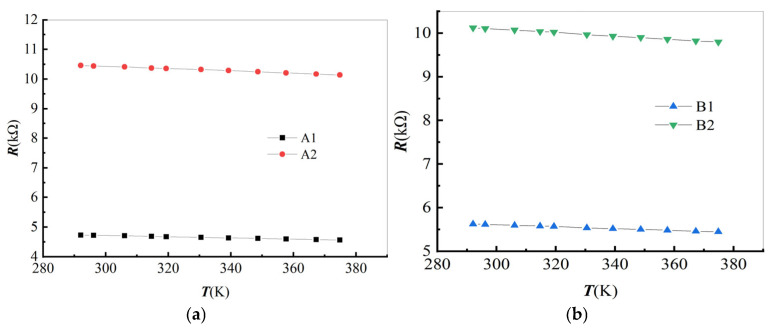
Temperature vs. resistance of a single carbon fiber: (**a**) A1 and A2 and (**b**) B1 and B2.

During the heating process, A1 and B1 are both in a straight-line shape, as in Figure 4a. A2 and B2 have curved shapes, as in Figure 4b. As temperature increases, the resistance of each specimen decreases, as shown in Figure 7.

#### 4.2.1. Linear Fitting of Temperature Resistance

The fitting results of the data in Figure 7 are shown in Table 4. According to these results, during the heating process the following is true: (1) As the temperature increases, the resistance of all specimens decreases, with correlation coefficient reaching 0.99. This indicates that carbon fiber is a Negative Temperature Coefficient (NTC) material. (2) Due to the constraints of the electrodes and substrate glass, the resistance change rate of A1 and B1 decreases. A1 decreased by 47.03% compared with A2. B1 has decreased by 45.75% compared to B2.

#### 4.2.2. Nonlinear Fitting of Temperature–Conductance

**Figure 8:**
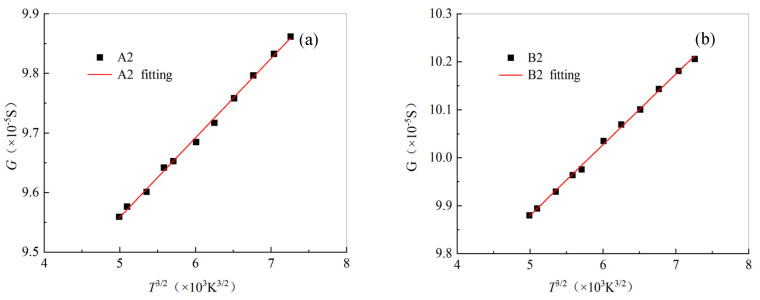
Temperature vs. conductance of the single carbon fiber specimens: (**a**) A2; (**b**) B2.

A2 and B2 remained in a curved state throughout the entire process and were not subjected to axial tension during the heating process. As shown in Figure 8, the conductance of A2 and B2 follows a linear relationship with temperature, according to Equation (11), and the corresponding fitting parameters are shown in Table 5. This result is consistent with that in reference [24]. It should be noted that the analysis object in reference [24] is carbon fiber bundles. This indicates that the relationship between the electrical conductivity and temperature of a single carbon fiber and that of carbon fiber bundles is consistent. This also indicates that it is feasible to adopt Equation (9) in the theoretical analysis of the thermal resistance of a single carbon fiber.

#### 4.2.3. Two Types of Comparison of the Temperature-Resistance Fitting

The resistance of A2 can be expressed as Equations (25) and (26), according to Equation (11) and Table 4 and Table 5.

(25) R=11.593−0.00387T

(26) $$R=\frac{1000}{1.332T^{3/2}+88.93}$$

The resistance of B2 can be expressed as Equations (27) and (28), according to Equation (11) and Table 4 and Table 5.

(27) R=11.291−0.004T

(28) $$R=\frac{1000}{1.472T^{3/2}+91.44}$$

**Figure 9:**
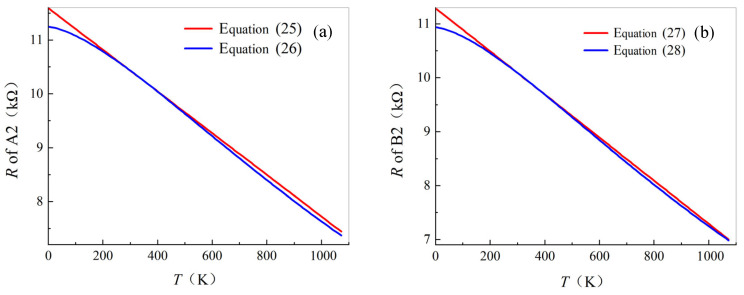
Theoretical analysis of temperature vs. resistance: (**a**) A2 and (**b**) B2.

Figure 9 can be obtained based on Equations (25)–(28). The relative errors of the two types fitting are both less than 0.2%, within 223–473 K (Figure 9). The relative error between the two types of fitting is the smallest at 293K.

This result indicates that the resistance of a single carbon fiber (Model II) within the temperature range of 223 K to 473 K can be calculated by either the temperature-based linear model or the nonlinear model with extremely small error. Therefore, a single carbon fiber may serve as a temperature-sensing element in extreme environments.

### 4.3. Analysis of the Temperature-Resistance Sensitivity Coefficient

The resistance fitting values in Table 4 are revised to obtain Table 6 according to the initial conditions: *T*_0_ = 293 K, *F*_0_ = 0 N. Based on Table 6 and Equations (19)–(21), Table 7 is obtained. In Table 7, the GFs under temperature effect ($K_{S}^{T}$) of A2 and B2 are 925 and 1039, respectively. According to Table 1, the $K_{S}^{T}$ values of A2 and B2 are mainly determined by the electron mobility in the piezoresistive effect. When Δ*T* = 2.5 K, that is, when the axial contraction strain of A2 is 1 με, the resistance variation rate of A2 is 9.25$\;\times \;10^{-4}$.

### 4.4. Analysis of the Strain Resistance Sensitivity Coefficient

**Figure 10:**
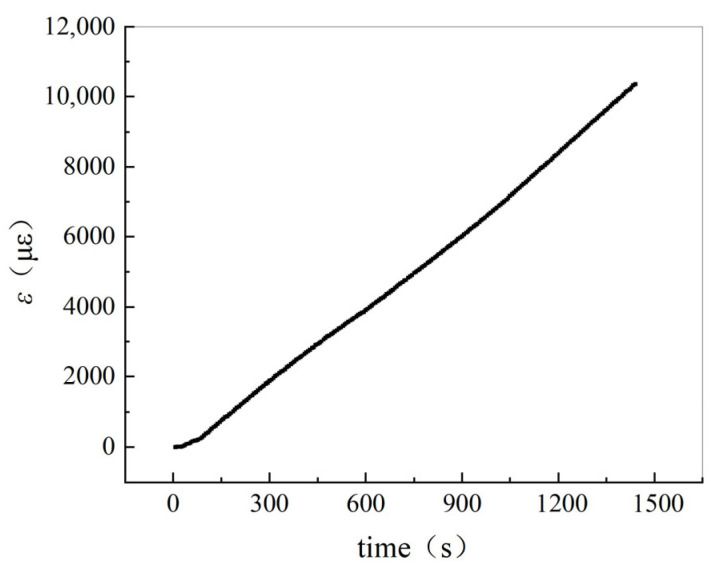
Strain measured on the tensile side of the four-point-bending specimen.

**Figure 11:**
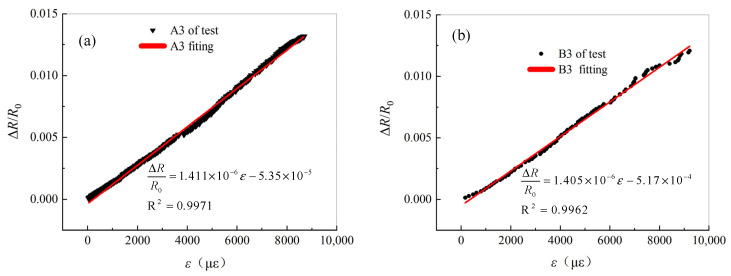
Strain vs. resistance change rate of the four-point-bending specimen: (**a**) A3 and (**b**) B3.

The strain on the tensile side of the four-point bending specimen is shown in Figure 10, with the corresponding loading rate of 0.5 mm/min. The strain resistance change rate and fitting results of a single carbon fiber filament on the tensile side are shown in Figure 11. According to this result, the resistance change rate of a single carbon fiber filament increases linearly with the increase of tensile strain, and the linear correlation is above 99.6%. The values of $K_{S}^{F}$ are 1.411 for A3 and 1.405 for B3. A3 and B3 are the single carbon fiber specimens for the four-point bend test.

According to Equation (8) and Table 1, the analysis results of A3 and B3, and the results from reference [27] are shown in Table 8. In reference [27], the proportion of the piezoresistive effect is 13.4%, and its impact cannot be ignored. The axial piezoresistivity $\pi_{f}$ of semiconductor materials at room temperature is 40–80$\;\times \;10^{-11}$$\;m^{2}/N$. Hence, carbon fiber differs significantly from semiconductors. The analysis results of A3 and B3 in Table 8 also support this conclusion. Compared with reference [27], the absolute values of the axial piezoresistivity of A3 and B3 are lower. The main reason for this is that the graphitization degree of the carbon fibers produced in A3 and B3 is higher. Furthermore, the Young’s modulus of A3 and B3 are relatively high, with the corresponding $K_{S}^{F}$ values of 1.411 and 1.405, respectively, both of which are greater than strain resistance sensitivity coefficient reported in reference [27]. The force-resistance sensitivity coefficient of the resistance strain gauge based on copper oxide is approximately 2.0. The four-point bend test analysis indicates that the force resistance sensitivity coefficients of A3 and B3 are lower than those of the Ni–Cu-based resistance strain gauges.

## 5. Conclusions

This study analyzes the conductive model and resistance theory of carbon fibers. Based on the resistance theory analysis of a single carbon fiber, theoretical models under different effects are proposed. According to the quasi-static equivalent superposition theory, the theoretical model of the GF of a single carbon fiber under the thermal–mechanical coupling effect was analyzed. Corresponding experiments were designed, and relevant tests and analyses were carried out. The main conclusions are as follows:

(1) The g-C_3_N_4_ in carbon fibers is a semiconductor, which leads to the semiconductive-like conductive properties of carbon fibers. The theoretical models of resistance and the GF of a single carbon fiber under different effects can reflect the influence of the piezoresistive effect and strain.
(2) Tests on single carbon fiber specimens with different initial states under the effect of temperature show that the strain sensitivity of T700S is higher than that of SYT49S. As the temperature rises, the resistance of single carbon fiber specimens with different initial states linearly decreases. The initial state of the carbon fiber specimen has significant impact on its resistance change rate.
(3) Within 223 K to 473 K, the resistance of free single carbon fibers can be calculated by linear model or nonlinear models, where the relative error between the two is less than 0.2%.
(4) The theoretical calculation results of the GF of single carbon fiber specimens under the coupling effect are consistent with the test fitting results, and the corresponding theoretical model is reliable. Free single carbon fiber is highly sensitive to the effect temperature, making it suitable as a temperature-sensing device.
(5) The four-point bending test analysis shows that the force resistance sensitivity coefficients of A3 and B3 are 1.411 and 1.405, respectively, which are significantly lower than those of the Ni–Cu-based resistance strain gauges. The graphitization degree of SYT49S is higher than that of T700S.

This study revealed that the reason why carbon fiber exhibits semiconductor-like conductive properties is due to the g-C_3_N_4_ in carbon fiber and gives the resistance model of a single carbon fiber. The theoretical model of the GF of a single carbon fiber established under the effects of strain, temperature, and thermal–mechanical coupling provided insights for the self-sensing and self-monitoring theoretical research of CFRPs. The thermal-resistance and force-resistance tests of a single carbon fiber were designed, and corresponding analyses were conducted based on the theoretical model. In addition, future work may consider wet–heat–aging coupling effects on carbon fibers or CFRPs in different service environments of various projects.

## Figures and Tables

**Table 1:** The GF of a single carbon fiber under typical effects.

Typical Effect	GF	Strain Effect	Piezoresistive Effect	
			Carrier Concentration	Electron Mobility
*F* ≠ 0 Δ*T* = 0	$K_{S}^{F}$	1+2μ	$\pi_{f}E$	0
*F* = 0 Δ*T* ≠ 0	$K_{S}^{T}$	$1+2\mu^{T}$	$1-2\mu^{T}$	$-\frac{3}{2\alpha }\frac{T^{1/2}}{T^{3/2}+\upsilon_{0}/c}$
*F* ≠ 0 Δ*T* ≠ 0	$K_{S}$	$(1+2\mu )\frac{\varepsilon^{F}}{\varepsilon }+(1+2\mu^{T})\frac{\varepsilon^{T}}{\varepsilon }$	$\pi_{f}E\frac{\varepsilon^{F}}{\varepsilon }+(1-2\mu^{T})\frac{\varepsilon^{T}}{\varepsilon }$	$-\frac{3}{2\alpha }\frac{T^{1/2}}{T^{3/2}+\upsilon_{0}/c}\cdot \frac{\varepsilon^{T}}{\varepsilon }$

**Table 2:** Parameters of the single carbon fiber used in this study.

Product Model	Tensile Strength (MPa)	Young’s Modulus *E* (GPa)	Elongation (%)	Poisson’s Ratio μ	Diameter *D* (μm)	Volume Resistivity ρ (×10^−5^ Ω·m)	Temperature Expansion Coefficient (×10^−6^ K^−1^)	
							Axial	Radial
SYT49S-12k	4900	240	2.0	0.30	7.0	1.5	−0.40	–
T700S-6k	4900	230	2.1	0.30	7.0	1.6	−0.38	27

**Table 3:** Parameters of the single carbon fiber specimen (*T*_0_ = 293 K).

Product Model	Specimen	Initial State	Model Type	Electrode Distance (mm)	Initial Resistance *R*_0_ (kΩ)	Initial Length *L*_0_ (mm)	Diameter *D* (μm)	Volume Resistivity ρ (×10^−5^ Ω·m)
SYT49S-12k	A1	Straight	I	13.34	4.731	13.34	7.0	1.36
	A2	Curved	II	–	10.461	29.50		
T700S-6k	B1	Straight	I	14.22	5.622	14.22	7.0	1.52
	B2	Curved	II	–	10.121	25.60		

Note: Model types are shown in Figure 4.

**Table 4:** Temperature vs. resistance fitting of the single carbon fiber specimens (*T*_0_ = 293 K).

Product Model	Specimen	Initial State	Mode Type	Effect	*L*_0_ (mm)	Resistance Fitting (Ω)	R^2^
SYT49S-12k	A1	Straight	I	Δ*T*, Δ*F*	13.34	R=5330−2.05T	0.9993
	A2	Curved	II	Δ*T*	29.50	R=11,593−3.87T	0.9972
T700S-6k	B1	Straight	I	Δ*T*, Δ*F*	14.22	R=6257−2.17T	0.9982
	B2	Curved	II	Δ*T*	25.60	R=11,291−4.0T	0.9984

Note: Δ*F* is caused by electrode constraints in A1 and B1.

**Table 5:** Temperature vs. resistance of the single carbon fiber specimens (*T*_0_ = 293 K).

Product Model	Specimen	State	Effect	*L*_0_ (mm)	Conductance (μS)	R^2^
SYT49S-12k	A2	Curved	Δ*T*	29.50	$G=1.332T^{3/2}+88.93$	0.9978
T700S-6k	B2	Curved	Δ*T*	25.60	$G=1.472T^{3/2}+91.44$	0.9984

**Table 6:** Temperature vs. resistance fitting of a single carbon fiber (*T*_0_ = 293 K).

Product Model	Specimen	Initial State	Model Type	Effects	Resistance Fitting (Ω)	R^2^
SYT49S-12k	A1	Straight	I	Δ*T*, Δ*F*	R=4729−2.05ΔT	0.9993
	A2	Curved	II	Δ*T*	R=10,459−3.87ΔT	0.9972
T700S-6k	B1	Straight	I	Δ*T*, Δ*F*	R=5621−2.17ΔT	0.9982
	B2	Curved	II	Δ*T*	R=10,119−4.00ΔT	0.9984

Note: $\Delta T=T-T_{0}$.

**Table 7:** GF of temperature vs. resistance of a single carbon fiber (*T*_0_ = 293 K, Δ*F* = 0).

Product Model	Specimen	Initial State	Model Type	Resistance Fitting (Ω)	$\mathrm{Resistance}\;\mathrm{Variation}\;\mathrm{Rate}\;\frac{\Delta R}{R_{0}}$ $(\times 10^{-4})$	α (×10^−6^ K^−1^)	$K_{S}^{T}$
SYT49S-12k	A2	Curved	II	R=10,459−3.87ΔT	−3.70ΔT	−0.40	925
T700S-6k	B2	Curved	II	R=10,119−4.00ΔT	−3.95ΔT	−0.38	1039

**Table 8:** Strain vs. resistance change rate analysis of the single carbon fiber specimen (four-point bend, 293 K).

Product Model	Specimen	Initial State	Effect	Strain Effect 1 + 2*μ*	Piezoresistive Effect *π*_f_ *E*	Axial Piezoresistivity *π*_f_ (m^2^/N)	$K_{S}^{F}$
SYT49S-12k	A3	Straight	Δ*F*	1.60	0.189	$7.875\times 10^{-11}$	1.411
T700S-6k	B3	Straight	Δ*F*	1.60	0.195	$8.478\times 10^{-11}$	1.405
Reference [27]		Straight	Δ*F*	1.56	0.21	$11.7\times 10^{-11}$	1.35

## Data Availability

The original contributions presented in this study are included in the article. Further inquiries can be directed to the corresponding author.
